# X-ray dark-field chest imaging for detection and quantification of emphysema in patients with chronic obstructive pulmonary disease: a diagnostic accuracy study

**DOI:** 10.1016/S2589-7500(21)00146-1

**Published:** 2021-10-25

**Authors:** Konstantin Willer, Alexander A Fingerle, Wolfgang Noichl, Fabio De Marco, Manuela Frank, Theresa Urban, Rafael Schick, Alex Gustschin, Bernhard Gleich, Julia Herzen, Thomas Koehler, Andre Yaroshenko, Thomas Pralow, Gregor S Zimmermann, Bernhard Renger, Andreas P Sauter, Daniela Pfeiffer, Marcus R Makowski, Ernst J Rummeny, Philippe A Grenier, Franz Pfeiffer

**Affiliations:** a Department of Physics, Technical University of Munich, Garching, Germany; b Munich School of BioEngineering, Technical University of Munich, Garching, Germany; c Institute for Advanced Study, Technical University of Munich, Garching, Germany; d Department of Diagnostic and Interventional Radiology, School of Medicine & Klinikum rechts der Isar, Technical University of Munich, Munich, Germany; e Department of Cardiology, Angiology, and Pneumology, School of Medicine & Klinikum rechts der Isar, Technical University of Munich, Munich, Germany; f Philips Research Hamburg, Hamburg, Germany; g Philips Medical Systems DMC Hamburg, Hamburg, Germany; h Department of Clinical Research and Innovation, Hôpital Foch, Suresnes, Paris, France

## Abstract

**Background:**

Although advanced medical imaging technologies give detailed diagnostic information, a low-dose, fast, and inexpensive option for early detection of respiratory diseases and follow-ups is still lacking. The novel method of x-ray dark-field chest imaging might fill this gap but has not yet been studied in living humans. Enabling the assessment of microstructural changes in lung parenchyma, this technique presents a more sensitive alternative to conventional chest x-rays, and yet requires only a fraction of the dose applied in CT. We studied the application of this technique to assess pulmonary emphysema in patients with chronic obstructive pulmonary disease (COPD).

**Methods:**

In this diagnostic accuracy study, we designed and built a novel dark-field chest x-ray system (Technical University of Munich, Munich, Germany)—which is also capable of simultaneously acquiring a conventional thorax radiograph (7 s, 0·035 mSv effective dose). Patients who had undergone a medically indicated chest CT were recruited from the department of Radiology and Pneumology of our site (Klinikum rechts der Isar, Technical University of Munich, Munich, Germany). Patients with pulmonary pathologies, or conditions other than COPD, that might influence lung parenchyma were excluded. For patients with different disease stages of pulmonary emphysema, x-ray dark-field images and CT images were acquired and visually assessed by five readers. Pulmonary function tests (spirometry and body plethysmography) were performed for every patient and for a subgroup of patients the measurement of diffusion capacity was performed. Individual patient datasets were statistically evaluated using correlation testing, rank-based analysis of variance, and pair-wise post-hoc comparison.

**Findings:**

Between October, 2018 and December, 2019 we enrolled 77 patients. Compared with CT-based parameters (quantitative emphysema ρ=–0·27, p=0·089 and visual emphysema ρ=–0·45, p=0·0028), the dark-field signal (ρ=0·62, p<0·0001) yields a stronger correlation with lung diffusion capacity in the evaluated cohort. Emphysema assessment based on dark-field chest x-ray features yields consistent conclusions with findings from visual CT image interpretation and shows improved diagnostic performance than conventional clinical tests characterising emphysema. Pair-wise comparison of corresponding test parameters between adjacent visual emphysema severity groups (CT-based, reference standard) showed higher effect sizes. The mean effect size over the group comparisons (absent–trace, trace–mild, mild–moderate, and moderate–confluent or advanced destructive visual emphysema grades) for the COPD assessment test score is 0·21, for forced expiratory volume in 1 s (FEV_1_)/functional vital capacity is 0·25, for FEV_1_% of predicted is 0·23, for residual volume % of predicted is 0·24, for CT emphysema index is 0·35, for dark-field signal homogeneity within lungs is 0·38, for dark-field signal texture within lungs is 0·38, and for dark-field-based emphysema severity is 0·42.

**Interpretation:**

X-ray dark-field chest imaging allows the diagnosis of pulmonary emphysema in patients with COPD because this technique provides relevant information representing the structural condition of lung parenchyma. This technique might offer a low radiation dose alternative to CT in COPD and potentially other lung disorders.

**Funding:**

European Research Council, Deutsche Forschungsgemeinschaft, Royal Philips, and Karlsruhe Nano Micro Facility.

## Introduction

Although there have been tremendous technological advances in diagnostic imaging systems, the assessment of pulmonary microstructural changes remains challenging in clinical routine. X-ray dark-field imaging was introduced into the laboratory setting in 2008;^1^ this technique delivers complementary imaging information on the lung's microstructure beyond conventional chest x-rays.^2^, ^3^, ^4^ By contrast to common x-ray imaging, which measures the attenuation of x-rays, dark-field contrast is related to coherent small-angle scattering of x-rays at microscopic structures within the specimen.^1^, ^5^

**Research in context**

**Evidence before this study**

We searched PubMed for articles published between database inception and March 15, 2021, using the search terms “x-ray dark-field” and “emphysema”. No language restrictions were applied. The search yielded 11 pre-clinical (small-animal, ex-vivo tissue, and cadaver) studies, which are mostly based on artificially induced disease models. The main outcome of these studies is a high detection sensitivity of the dark-field method with respect to structural changes associated with pulmonary emphysema. Studies based in living humans were not identified in our search.

**Added value of this study**

We present the first x-ray dark-field chest images of human participants in vivo and show the feasibility of the method in a clinical setting. We conceived, constructed, and commissioned a custom-built first demonstrator system suitable for patient use. This system satisfies clinical demands regarding safety, usability, acquisition time, radiation dose, field of view, and image quality. This study marks the transition from investigating the use of x-ray dark-field radiography in disease models to evaluating the actual diagnostic performance in patients.

**Implications of all the available evidence**

Our findings indicate that x-ray dark-field radiography provides image-type information of the lungs’ underlying microstructure in humans. In view of the strong link between alveolar structure and the functional condition of the lung, this capability is highly relevant for respiratory medicine and might help to establish a better understanding of pulmonary disorders. For the early detection of chronic obstructive pulmonary disease, which is generally accompanied by structural impairments of the lung, this novel technique might support resolving the prevalent underdiagnosis reported in literature. The effective dose of dark-field radiography is substantially lower (by about 100 times) than that of thorax CT, indicating that dark-field radiography could be used as a broadly deployed screening tool.

The lung is composed of very fine interfaces between air and soft tissue to maximise the surface to volume ratio, which is essential for an efficient gas exchange. Repeated refraction and scattering of incident x-rays at these interfaces induces a high dark-field signal, which gives great potential to x-ray dark-field pulmonary imaging.^2^, ^3^, ^4^ Pulmonary disorders such as emphysema, fibrosis, lung cancer, or pneumonia have direct effect on the structural properties of the lung by degrading, condensing, displacing, or infiltrating lung tissue, which leads to an alteration of the dark-field signal. In these and other conditions, x-ray dark-field imaging has shown to be a diagnostic improvement over conventional chest radiography in small-animal studies.^6^, ^7^, ^8^, ^9^, ^10^, ^11^, ^12^

Chronic obstructive pulmonary disease (COPD) is a major medical condition and is the cause of millions of deaths every year worldwide.^13^ COPD is induced by chronic inflammation of lung tissue and manifests with irreversible airflow limitations. Pulmonary emphysema is one component of COPD, and is characterised by permanent dilation of air spaces and destruction of their walls distal to terminal bronchioles. CT is a validated imaging technique to visually and quantitatively assess the presence, extent, and pattern of emphysema in vivo.^14^ However, chest CT is currently not considered as a standard of care in the diagnosis and management of mild to moderate COPD.^15^ The suitability of CT is restricted because of the high radiation exposure of about 7 mSv for a regular chest examination. ^16^ Even if low-dose techniques are applied, the radiation exposure is still in the range of 2 mSv.^17^

A chest x-ray is usually the first diagnostic tool used in the evaluation of the patient's lungs, because it is fast, accessible, and inexpensive. The applied radiation dose is comparably low with an average effective dose of about 0·02 mSv for a single posterior–anterior image.^16^ However, it is well documented that plain chest radiography has low sensitivity for detecting emphysema.^18^

Because of its high sensitivity to microstructural parameters, the question arises whether the novel dark-field technique might offer an alternative low-dose imaging approach that allows an improved medical assessment of the lungs. The aim of this work is to show the application of dark-field chest radiography in living humans and to highlight the technique's future potential for diagnostic imaging of human lungs. We selected pulmonary emphysema in patients with COPD as the focus of this initial experimental trial.

## Methods

### Patients

Between October, 2018 and December, 2019 a total of 77 patients were included prospectively in this diagnostic accuracy study of x-ray dark-field chest imaging. All participants gave written informed consent and were approached after undergoing a medically indicated chest CT examination that was part of personal medical procedures not related to the study. Recruitment was performed among tumour follow-up examinations at the Department of Radiology and Pneumology of our site (Klinikum rechts der Isar, Technical University of Munich, Munich, Germany).

Patients with either healthy lungs or signs of emphysematous impairment according to CT were included. Patients with pulmonary pathologies or conditions other than COPD, that might influence lung parenchyma (eg, pulmonary congestion or lung cancer), were excluded.

To investigate the performance of x-ray dark-field chest imaging for the detection of early stages of COPD, the focus in the disease group was on patients with initial indications of emphysema revealed in CT, but still absent obstruction according to pulmonary function testing (ratio of forced expiratory volume in 1 s [FEV_1_] and functional vital capacity [FVC] higher than 0·7).^19^ However, moderate to severe stages of emphysema were also included to obtain an impression of the full signal range of dark-field signal across the entire spectrum of emphysema severity.

This study was approved by the institutional ethics review board and the German Federal Office for Radiation Protection (Bundesamt für Strahlenschutz).

### Procedures

**Figure 1:**
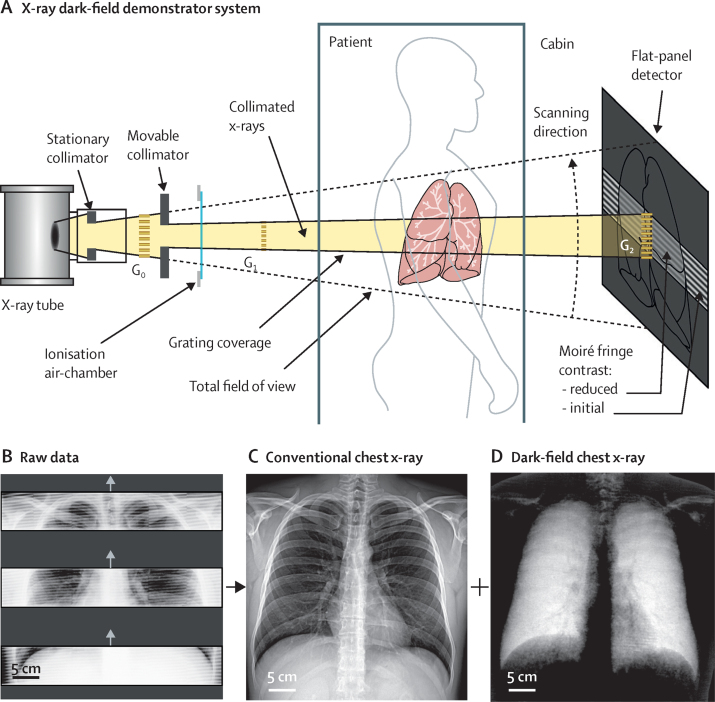
Schematic of the x-ray dark-field demonstrator system (A) and examples of raw detector imaging data (B), retrieved conventional chest x-rays (C), and dark-field chest x-rays (D) from the first in-human application (A) The moiré fringe pattern, which is generated by the interplay between the phase grating G_1_ and the analyser grating G_2_, is scanned across the patient's thorax. Subsequent processing of the image stack analyses the changes in the moiré fringe pattern and retrieves conventional and dark-field radiographs based on attenuation and small-angle scattering of x-rays within the patient's thorax. Behind regions with a high fluctuation of the refractive index (eg, alveolar microstructure), the contrast of the moiré pattern is reduced because of small-angle scattering. The G_0_ grating allows the use of standard x-ray source technology with a large focal spot. (B) Three (out of 195) example raw-data frames recorded during one scan of a healthy patient (male patient, aged 33 years, body-mass index 30·2 kg/m^2^, without lung disorders). The moiré pattern is clearly visible, superimposed onto the patient's thorax, which is subsequently used to retrieve a conventional attenuation chest radiograph (C) and the novel dark-field chest radiograph (D). Intact alveolar structure with a high density of air–tissue interfaces induces strong small-angle scattering, resulting in a pronounced dark-field signal within the lung region. The magnitude of the signal correlates with the number of interfaces^7^ and thus provides structural information on alveoli that are typically smaller than the resolution limit of the imaging system. Key system specifications for the demonstrator system are a field of view of 37 × 37 cm^2^ (patient plane), effective radiation dose of 0·035 mSv (for a male weighing 73 kg), and an acquisition time of 7 s.

A clinical demonstrator system capable of acquiring both dark-field and attenuation x-ray images of an entire human thorax in about 7 s scan time was conceived, constructed, commissioned, and integrated in the clinical infrastructure of our site (Department of Radiology, Klinikum rechts der Isar; figure 1A). The present implementation of the system represents the final fusion of several important technological developments, which have been pursued in multiple preparatory research projects leading up to this work.^1^, ^4^, ^20^, ^21^, ^22^, ^23^, ^24^, ^25^, ^26^ The system's design is based on standard medical components, such as x-ray source, collimator, and flat-panel detector, which are combined with a three-grating Talbot-Lau x-ray interferometer.^1^, ^20^ An example of raw data frames and retrieved attenuation-based radiographs and dark-field-based radiographs from the examination of a patient without pulmonary disorders is shown in figure 1B–D.

Lungs appear radiolucent in the conventional image, primarily related to their high air content. In the dark-field image by contrast, a distinct and homogeneous signal from the lung is obtained. Intact alveolar structure with numerous air-tissue interfaces induces strong small-angle scattering, resulting in a highlighted depiction of the lungs that allows for an unimpeded assessment.

The effective radiation dose was determined to be 0·035 mSv using an anthropomorphic thorax phantom (ATOM 701, CIRS, USA, adult male, reference height of 173 cm and weight of 73 kg) with embedded thermoluminescent dosimeters. Further information about underlying principles and implementation of the demonstrator system is available in the appendix (pp 1–3) and a sequence of the scan is available in the video.

All participants underwent lung function testing according to the European Respiratory Society recommendations.^27^, ^28^ These tests consist of a combination of spirometry with body plethysmography (MasterScreen Body, Jaeger, Germany). For 42 patients, the individual diffusion capacity of the lung for carbon monoxide uptake during one single breath (DLCO SB) was available (MasterScreen PFT, Jaeger, Germany); no further specifying inclusion criteria were applied for this measure. COPD classification was based on post bronchodilator FEV_1_ as proposed by the Global Initiative for Chronic Obstructive Lung Disease (GOLD; no COPD or no obstruction was diagnosed for FEV_1_/FVC>0·7 in the measurement before bronchodilation; GOLD I was diagnosed if post bronchodilation FEV_1_ ≥80%, GOLD II if 50–79%, GOLD III if 30–49%, and GOLD IV if <30%).^19^ Clinical symptoms were evaluated using a COPD Assessment Test (CAT; GlaxoSmithKline, UK).

Imaging was done on a CT scanner (IQon Spectral CT, Royal Philips, Netherlands) during clinical routine using a standard clinical chest protocol (120 kV_p_, automatic angular tube current modulation). After a volumetric acquisition over the entire chest at full suspended inspiration, 0·9 mm-thick axial slices were reconstructed with high resolution for visual assessment and soft tissue algorithms for quantitative assessment (iDose, Royal Philips, Netherlands). The vendor-specific noise suppression level 6 (iDose 6) was applied. Quantitative analysis of emphysema was done by thresholding lung density at – 950 HU using commercially available software (IntelliSpace, Royal Philips, Netherlands) to obtain the CT emphysema index (ratio between emphysema and lung volume). According to the Fleischner Society, CT emphysema index values below 6% were not considered to represent clinically significant emphysema.^29^ Intravenous contrast material (iodine) was present for all 77 patients because recruitment was from cancer treatment follow-ups.

CT, pulmonary function testing, and dark-field examination were done on the same day for most patients. Maximum temporal distance between examinations was 7 days.

Visual emphysema assessment based on CT images was done by three trained radiologists (AAF, APS, and Jannis Bodden) with 4–12 years of lung imaging experience. As a scale, the Fleischner Society classification scheme^29^ was applied, grading emphysema severity in to the following group*s*: absent, trace, mild, moderate, confluent, and advanced destructive. Resulting group assignment was used as diagnostic reference standard for the individual patient's emphysema state in the statistical tests.

On the basis of the dark-field chest images, five readers (AAF, APS, DP, Jannis Bodden, and Florian Gassert) graded emphysema severity for each patient on a five-point Likert scale with the levels being no evidence of emphysema (0), beginning of emphysema (1), mild emphysema (2), moderate emphysema (3), and severe emphysema (4). The reader's diagnostic confidence regarding this severity rating was also collected (very uncertain [1], uncertain [2], certain [3], and very certain [4]). Additionally, dark-field signal intensity and homogeneity was rated within upper, middle, and lower subregions of the left lung and right lung. The signal intensity was graded on a seven-point Likert scale ranging from 0 (no signal) to 6 (maximum signal). Signal homogeneity was rated applying a four-point Likert scale with the levels being very inhomogeneous (1), moderately inhomogeneous (2), mildly inhomogeneous or mostly homogeneous (3), and homogeneous (4). Additionally, in the case of a homogeneity rating other than homogeneous, areal signal texture was graded by assigning the levels no texture (0), subtle stains smaller than 5 mm (1), medium stains of 5–10 mm (2), and large stains bigger than 10 mm (3).

All readers received training on grading dark-field images before the study evaluation. With a temporal distance of approximately 1 month to the actual assessment, dark-field images of a small patient subset (n=15) including all occurring emphysema severity levels were presented to the readers along with corresponding information on emphysema stage obtained from CT. During the training, the readers graded the dark-field images in consensus reading to establish the visual grading scale described earlier.

The three readers who did the visual CT assessment were also involved in the assessment of the dark-field images. To reduce this potential source of bias, CT and dark-field reading sessions were temporally decoupled (around 1 month) and patient order was randomised. Revision and adaptation of previous grading decisions were allowed during readings. Readers graded images independently and were masked to other clinical measures and had no additional information beyond the evaluated images.

### Statistical analysis

The Likert scales used for feature grading in the dark-field image readings were considered as interval-scaled, to obtain refinement between the grades by calculating the mean across all of the readers.

To assess the consistency of visual signal grading, Spearman rank correlation coefficients were calculated between the rated intensity levels and the mean dark-field signal measured over all image pixels included in the respective subregions. A description of the signal measurement is provided in the appendix (p 5).

To investigate how the functional condition of the lung is represented by CT and dark field, Spearman correlation tests with diffusion capacity (DLCO SB) and FEV_1_/FVC ratio from pulmonary function testing were conducted. As test parameters, quantitative emphysema index and visual emphysema grade (reader median) both derived from CT and dark-field signal rating were used. To obtain a single-value measure that describes the dark-field signal strength for each patient's lungs, the mean rated signal level over all readers and subregions (upper left, middle left, lower left, upper right, middle right, and lower right) was calculated. Correlations were considered significant if p<0·05.

For the assessment of the clinical performance of the dark-field chest images, the patients were grouped by Fleischner grade (reader median), serving as diagnostic reference for pulmonary emphysema. The confluent and advanced destructive groups were combined in the evaluations to achieve a sufficient sample size. To compare diagnostic accuracy, descriptive statistics within these groups were calculated for conventional clinical measures and for image features derived from the dark-field readings. Additionally, parameter variation over all groups was evaluated using the Kruskall-Wallis test (critical value at H=9·49 for a 95% confidence level) and pair-wise variation between adjacent groups were evaluated using the Mann-Whitney *U* test (difference was considered significant if p<0·05; effect size r=|z × n^−½^|; with r≥0·1 indicating a weak effect, r≥0·3 indicating a moderate effect, and r≥0·5 indicating a strong effect). The parameter for dark-field-based emphysema severity was obtained by calculating the average rating from all five readers. For the signal homogeneity and texture level, the mean over all six subregions and all readers was calculated. Open-source software frameworks (SciPy, Pandas, Python) were used for the calculations.

### Role of the funding source

Authors who are employees of Royal Philips were involved in developing hardware and control software of the demonstrator system, and data processing algorithms. None of the other funders of the study had any role in study design, data collection, data analysis, data interpretation, or writing of the report.

## Results

**Figure 2:**
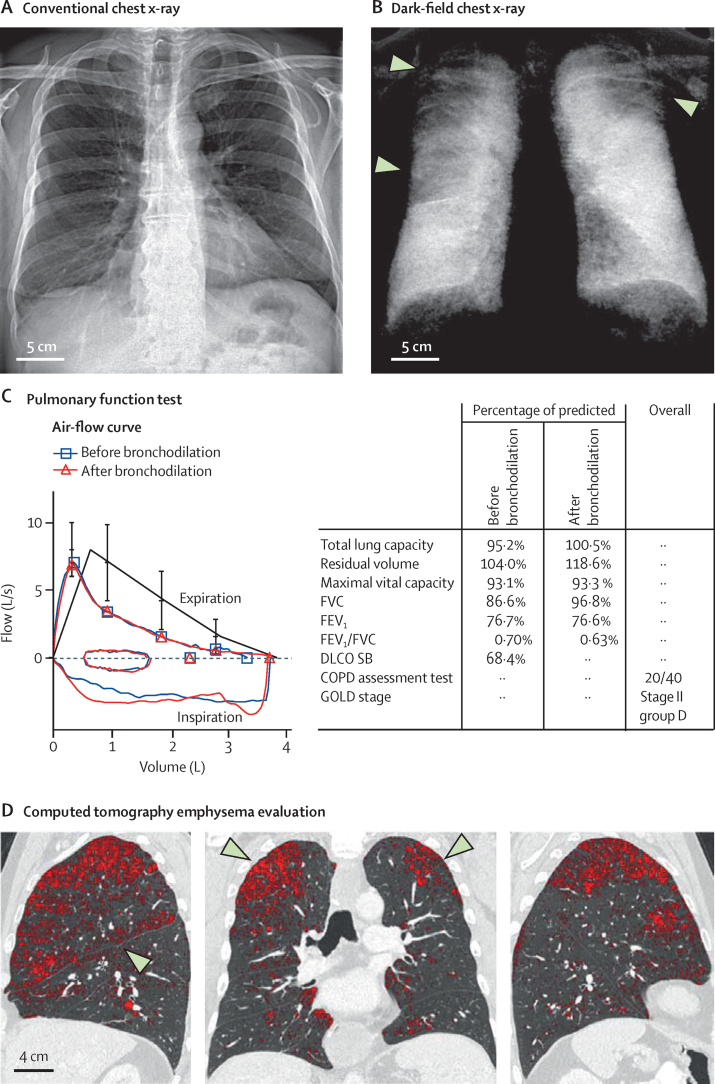
Conventional chest x-rays, dark-field chest X-rays, CT, and pulmonary function testing for an example patient (male, aged 58 years, body-mass index 27·6 kg/m^2^, 35 pack-years) Conventional (A) and dark-field (B) chest x-rays of a patient with localised emphysema, recorded with our demonstrator system. In the dark-field image (B), affected regions appear dark, indicating reduced small-angle scattering due to fewer air-tissue interfaces. By contrast, the attenuation image (A) is very limited in visualising the circumscribed emphysematous changes. Pulmonary function test (C) yields moderate obstruction still remaining post bronchodilation. Each lung function parameter is normalised with respect to the expected value of a patient without pulmonary disorders having same age, sex, and height. CT densitometry yields an emphysema index of 11·5% implying this fraction of the lung volume has density values below –950 HU. The corresponding voxels in the CT slices (D; lung parenchymal window settings applied) are shown in red. Dark-field and CT images yield consistent information regarding parenchymal condition. COPD=chronic obstructive pulmonary disease. DLCO SB=diffusion capacity of the lung for carbon monoxide in one single breath. FEV_1_=forced expiratory volume in 1 s. FVC=forced vital capacity. GOLD=Global Initiative for Chronic Obstructive Lung Disease.

An overview of the study population is shown in table 1. Figure 2 shows the types of data generated during the study, using an example male patient (aged 58 years, body-mass index [BMI] 27·6 kg/m^2^, 35 pack-years of smoking). The conventional attenuation image (figure 2A) shows no conspicuous features suggesting structural impairments of the lung. Most parts of the lung yield dark-field values (figure 2B) comparable to the ones of the healthy lung in figure 1D. However, in the apical area of the right and left lung, spreading over approximately 6– 7 cm in caudal direction, the signal is strongly decreased. Additionally, in the middle right zone, a moderate reduction is recognisable reaching across the prominent interface between upper and middle lobe. These observations are consistent with this patient's mean reader ratings of the dark-field signal intensity (upper right 1·8 [SD 0·8], middle right 2·8 [0·8], lower right 4·6 [0·5], upper left 2·0 [0·7], middle left 5·0 [0·7], and lower left 4·8 [0·4]), signal homogeneity (upper right 1·4 [0·9], middle right 1·4 [0·9], lower right 2·8 [0·8], upper left 1·4 [0·9], middle left 2·8 [0·8], and lower left 2·8 [0·8]), and areal signal texture (upper right 2·8 [0·4], middle right 2·8 [0·4], lower right 1·6 [1·3], upper left 2·8 [0·4], middle left 1·2 [1·1], and lower left 1·2 [1·1]). Based on the dark-field image, the mean emphysema severity was graded between moderate and severe (3·6 [0·5]). The air-flow curve (figure 2C) shows a characteristic concavity in the descending limb indicating obstructed airways.^30^ Except for FEV_1_, parameters from pulmonary function test (figure 2C) are deviating moderately from predicted values after administration of a bronchodilator. The FEV_1_/FVC ratio lies just at the abnormality threshold (FEV_1_/FVC=0·70, before bronchodilation) applied in the GOLD classification scheme.^19^ The decreased FEV_1_ that remains after bronchodilation indicates irreversible airflow impairment. The decreased DLCO SB indicates reduced alveolar surface. In figure 2D, coronal and sagittal CT slices of the thorax are shown. 11·5% of all voxels assigned to the lung are classified as emphysematous, yielding density values lower than –950 HU. Affected regions (marked in red) coincide with those of decreased dark-field signal. In the right lung, highlighted voxels align along the inclined lobe interface, matching the region with the moderately decreased dark-field signal. By contrast, regions featuring higher density values coincide with regions of stronger small-angle scattering, indicating an intact alveolar structure in both methods, respectively. Visual CT assessment based on the Fleischner scheme indicated confluent emphysema (median 4 [IQR 0·0] over readers) for this patient's lungs.

**Table 1:** Study population

		**Study population (n=77)**
Sex		
	Female	30 (39%)
	Male	47 (61%)
Ethnicity		
	White	77 (100%)
Age, years		
	Female	63·3 (11·3; 42–80)
	Male	65·9 (12·7; 30–91)
	Combined	64·9 (12·1; 30–91)
Body-mass index, kg/m^2^		26·0 (4·7; 16·4–38·9)
GOLD stage		
	Stage 0	64 (83%)
	Stage I	2 (3%)
	Stage II	6 (8%)
	Stage III	3 (4%)
	Stage IV	2 (3%)

Data are n (%) or mean (SD; range). GOLD=Global Initiative for Chronic Obstructive Lung Disease.

**Figure 3:**
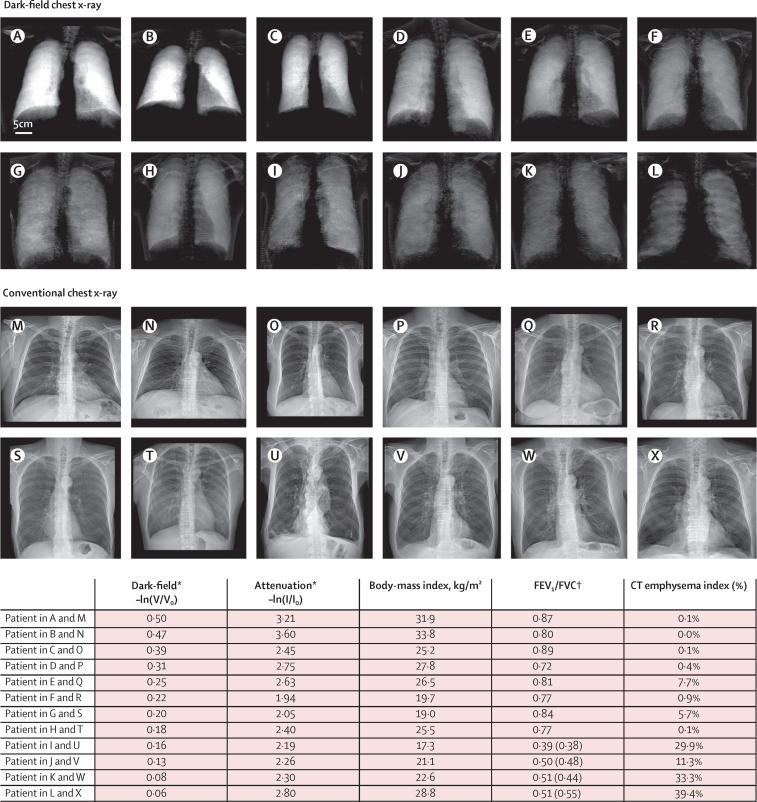
Dark-field chest x-rays, conventional chest x-rays, spirometry, and CT findings for patients with increasing severity of chronic obstructive pulmonary disease In the dark-field images (A–L), a gradual decrease in signal strength, ranging from no pulmonary disorder to severe emphysema, is shown. Additionally, local signal variations can be recognised. With a decline in the number of alveolar interfaces, less small-angle scattering is induced, resulting in decreasing dark-field signal values. No significant variation in contrast of the lung relative to surrounding features is recognisable in the respective attenuation images (M–X). The ribcage, superimposing the lungs in the attenuation images, is barely present in the dark-field images. The dark-field and attenuation pairs A-M, B-N, C-O, D-P, E-Q, F-R, G-S, H-T, I-U, J-V, K-W, and L-X correspond to the same patient. Within each of the contrast channels, identical window and level settings are applied. The scale bar in (A) applies for all panels. FEV_1_=forced expiratory volume in 1 s. FVC=forced vital capacity. *Measurement of dark-field and attenuation values (negative logarithm of normalised visibility [V/V_0_] and normalised intensity [I/I_0_, transmission]) was obtained by manually selecting the outline of the lung in the attenuation image and calculating the mean signal value over all included pixels. In the attenuation image, the value extraction was done before the image post-processing. †Ratio as obtained from pulmonary function testing. For patients exhibiting a ratio below 0·7, a second measurement with bronchodilator was taken. The bronchodilator results are shown in parentheses.

In figure 3, dark-field images (A–L) of 12 patients of our study with gradually decreasing signal intensity in the pulmonary region are lined up with the corresponding attenuation images (M–X). The associated BMI, FEV_1_/FVC ratio, CT emphysema index, and the average dark-field and attenuation values measured within the lung region are also shown in figure 3. In this group, a decreasing dark-field signal is accompanied by decreasing pulmonary function and increasing CT emphysema index, which are characteristic indications for a reduction of the alveolar surface. In the attenuation images, the observed variation in dark-field signal is not accompanied by notable contrast variations of the lung with respect to the surrounding area. The average signal within the lungs is related to the collective attenuation by all materials overlaying the lungs, in particular muscle, fatty tissue, and breast tissue, which is reflected by a very strong linear correlation between mean attenuation signal and BMI (r=0·84, p<0·0001, n=77). Apart from effects such as Compton scattering and beam-hardening, which are largely correctable, the dark-field signal is not affected by microscopically uniform and thus non-scattering material superimposing the lungs. Therefore, there is a weak linear correlation between the mean dark-field signal and BMI (r=0·34, p=0·0028, n=77). Although the dark-field images exhibit a gradual signal decrease within the lungs, inconspicuous findings for the FEV_1_/FVC ratio (eight patients with FEV_1_/FVC>0·7)^19^ and emphysema index (seven patients with CT emphysema index <6%)^29^ were found among these 12 examples.

Indicating a good agreement of objective image information and averaged reader grading, strong positive correlations were found between measured and rated dark-field signal intensity for all subregions (upper right 0·76, middle right 0·86, lower right 0·87, upper left 0·75, middle left 0·84, lower left 0·85, all p<0·0001, n=77).

Visual emphysema grading based on the CT images showed that 35 (45%) of 77 patients had absent emphysema, 19 (25%) had trace emphysema, nine (12%) had mild emphysema, seven (9%) had moderate emphysema, six (8%) had confluent emphysema, and one (1%) had advanced destructive emphysema.

**Figure 4:**
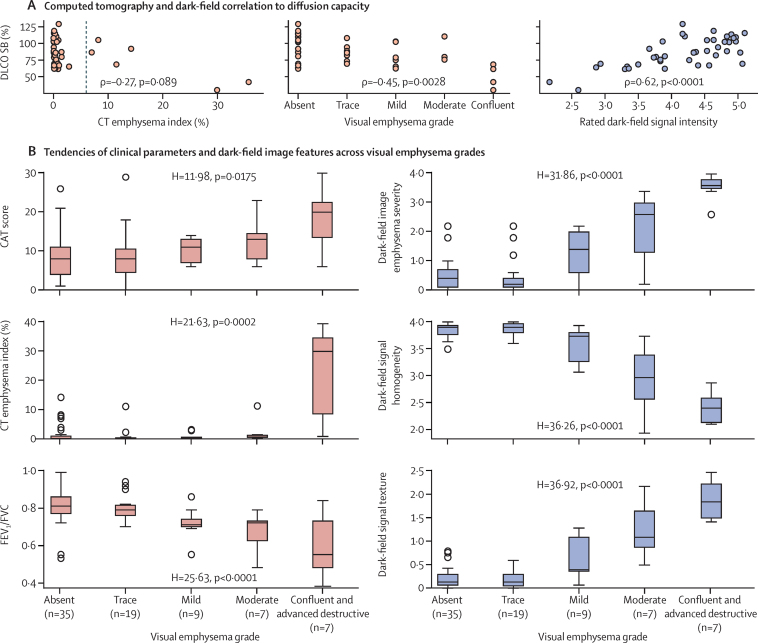
Scatter plots showing diffusion capacity versus CT and dark-field parameters and box plots showing clinical and dark-field image features across CT-based visually determined emphysema severity groups (A) Scatter plots show the respective correlations of the CT emphysema index, CT-based visual emphysema grade, and rated dark-field signal intensity with the DLCO SB. For DLCO SB, values are given with respect to the expected value of a patient without pulmonary disorders having same age, sex, and height. Visual emphysema grades correspond to the median rating of the three readers. Values for rated dark-field signal are means over all six subregions and all five readers. The strong link between diffusion capacity and structural lung condition is reflected in the good correlation with the dark-field signal. DLCO SB describes the condition of the respiratory surface with the ability to perform gas exchange and the dark-field signal describes the condition of the respiratory surface via small-angle scattering at microscopic interfaces. (B) Box plots show the tendencies found for the CAT score, CT emphysema index, FEV_1_/FVC ratio, dark-field-based emphysema rating, and dark-field image features across the Fleischner emphysema grades of the evaluated patients. If FEV_1_/FVC was less than 0·7, the measurement after bronchodilation was used for the FEV_1_/FVC ratio in the evaluation. The parameter for dark-field-based emphysema severity corresponds to the mean over all five reader ratings. To obtain parameters for homogeneity and texture level, the average over all six subregions and all readers was calculated. The Fleischner grade is assigned based on the size and distribution of regional density variations induced by parenchymal impairments. The associated alteration of alveolar microstructure also induces local signal variations in the dark-field image due to reduced small-angle scattering in affected regions. Consequently, a decreasing signal homogeneity and increasing texture is observable in the dark-field images with increasing visual emphysema grade. CAT=chronic obstructive pulmonary disease assessment test. DLCO SB=diffusion capacity of the lung for carbon monoxide in one single breath. FEV_1_=forced expiratory volume in 1 s. FVC=forced vital capacity.

Figure 4A shows scatter plots of DLCO SB versus CT emphysema index, visual emphysema grading, and rated dark-field signal intensity for all patients who underwent an examination of the diffusion capacity (n=42). The dark-field signal gives a stronger correlation (ρ=0·62, p<0·0001) than the CT emphysema index (ρ=–0·27, p=0·089) and the visual emphysema grading based on the CT images (ρ=–0·45, p=0·0028). For only the patients in the absent emphysema, trace emphysema, and mild emphysema Fleischner groups (n=35), for these correlation pairs we found no significant correlation for the CT parameters (ρ=–0·07, p=0·70 for CT emphysema index and ρ=–0·31, p=0·068 for visual emphysema grading) and a moderate correlation for the dark-field signal (ρ=0·46, p=0·0056). Additionally, we found moderate correlations for CT emphysema index (ρ=–0·52, p<0·0001) visual emphysema grading (ρ=–0·53, p<0·0001), and rated dark-field signal (ρ=0·49, p<0·0001) with FEV_1_/FVC (n=77).

An overview of the study results are shown in Table 2, Table 3. Rank-based analysis of variance indicates significant value variation across the Fleischner grades for all evaluated parameters, and conventional clinical and dark-field-based emphysema measures (table 3). Post-hoc pair-wise comparison of adjacent groups (absent–trace, trace–mild, mild–moderate, moderate–confluent or advanced destructive), however, yields improved discriminability for the visual features in the dark-field images compared with the conventional measures for emphysema diagnosis. None of the adjacent group pairs showed significant differences in the chronic obstructive pulmonary disease assessment test score. For the parameters from pulmonary function testing, we found one group pair (trace–mild) with significant difference for the parameters FEV_1_/FVC and FEV_1_ (% of predicted) and two group pairs (trace–mild and mild–moderate) for the residual volume (% of predicted). The CT emphysema index yields two distinguishable group pairs (trace–mild and moderate–confluent or advanced destructive). For the parameters signal homogeneity and areal texture retrieved from the reader study of the dark-field images, we obtain three group pairs (trace–mild, mild–moderate, and moderate–confluent or advanced destructive) which indicated significant variation. None of the evaluated parameters showed significant distinguishability for the absent–trace group pair (table 3; figure 4).

**Table 2:** Overall study results

	**Mean (SD; 95% CI)**	**Range**
**Clinical parameters**		
COPD assessment test score	10·0 (6·5; 8·5–11·4)	0·0–30·0
FEV_1_/FVC*	0·77 (0·12; 0·74–0·79)	0·38–0·99
FEV_1_ % of predicted*	96·3 (26·1; 90·3–102·2)	23·5–147·8
Residual volume % of predicted*	120·1 (41·7; 110·7–129·6)	55·2–231·9
DLCO SB % of predicted (N=42)	86·5 (21·7; 79·7–93·2)	30·0–129·1
CT emphysema index, %†	3·3 (8·0; 1·4–5·1)	0·0–39·4
Dark-field* −ln (*V*/*V*_0_)‡	0·29 (0·08; 0·27–0·31)	0·06–0·50
**Dark-field-based reader ratings**§		
Rated dark-field signal	4·21 (0·86; 4·02–4·41)	1·83–5·80
Emphysema severity level	0·98 (1·16; 0·72–1·25)	0·00–4·00
Homogeneity level	3·61 (0·53; 3·49–3·73)	1·93–4·00
Texture level	0·50 (0·63; 0·36–0·65)	0·00–2·50

COPD=chronic obstructive pulmonary disease. DLCO SB=diffusion capacity of the lung for carbon monoxide in one single breath. FEV_1_=forced expiratory volume in 1 s. FVC=forced vital capacity.

* For patients with FEV_1_/FVC<0·7 the results from after bronchodilator administration were used for the statistical analysis.

† Percentage of the segmented lung that exhibits density values below −950 HU.

‡ Value for one patient corresponds to the mean signal value of all pixels within the lungs.

§ Emphysema severity rating was obtained by calculating the mean over all five readers. For the parameters rated dark-field signal, homogeneity level, and texture level, the average over all six subregions and all readers was used for the evaluation.

**Table 3:** Study results by visual emphysema grade

	**Visual emphysema grades**					H value*; p value	**Pair-wise comparison**†				
	Absent (n=35)	Trace (n=19)	Mild (n=9)	Moderate (n=7)	Confluent, advanced destructive (n=7)		Absent–trace, p value (effect size)	Trace–mild, p value (effect size)	Mild–moderate, p value (effect size)	Moderate– confluent, advanced destructive, p value (effect size)	Mean effect size across group comparisons
**Clinical parameters**											
COPD assessment test score	8·60 (5·88); 8·00 (7·00)	8·53 (6·59); 8·00 (6·00)	9·89 (3·14); 11·00 (6·00)	12·43 (5·80); 13·00 (6·50)	18·29 (7·93); 20·00 (9·00)	11·98; 0·017	0·46 (0·01)	0·13 (0·21)	0·14 (0·28)	0·11 (0·34)	0·21
FEV_1_/FVC‡	0·81 (0·09); 0·81 (0·09)	0·81 (0·07); 0·79 (0·05)	0·72 (0·08); 0·71 (0·04)	0·67 (0·11); 0·72 (0·10)	0·60 (0·17); 0·55 (0·25)	25·63; <0·0001	0·21 (0·11)	0·0027 (0·53)	0·34 (0·12)	0·22 (0·22)	0·25
FEV_1_ % of predicted‡	104·04 (23·43); 105·90 (23·40)	105·93 (17·83); 103·40 (20·80)	88·46 (19·34); 83·10 (23·20)	79·87 (27·47); 86·00 (25·65)	57·69 (23·12); 54·30 (37·20)	21·94; 0·0002	0·46 (0·01)	0·019 (0·40)	0·42 (0·07)	0·063 (0·43)	0·23
Residual volume, % of predicted‡	109·42 (37·60); 106·60 (35·55)	105·49 (22·20); 104·00 (22·90)	120·67 (28·31); 117·00 (37·50)	155·67 (44·36); 156·50 (63·65)	177·33 (53·51); 208·80 (84·30)	16·20; 0·0028	0·47 (0·01)	0·047 (0·32)	0·045 (0·44)	0·26 (0·19)	0·24
CT emphysema index, %§	1·49 (3·12); 0·13 (0·84)	0·93 (2·54); 0·15 (0·41)	0·95 (1·18); 0·43 (0·28)	2·23 (4·00); 0·64 (0·76)	22·34 (15·84); 29·94 (25·93)	21·63; 0·0002	0·25 (0·09)	0·038 (0·34)	0·14 (0·28)	0·0053 (0·70)	0·35
**Dark-field-based reader evaluation**¶											
Emphysema severity level‖	0·47 (0·50); 0·40 (0·60)	0·45 (0·62); 0·20 (0·30)	1·24 (0·80); 1·40 (1·40)	2·11 (1·30); 2·60 (1·70)	3·54 (0·46); 3·60 (0·30)	31·86; <0·0001	0·27 (0·08)	0·0048 (0·49)	0·061 (0·40)	0·0051 (0·70)	0·42
Homogeneity level**	3·85 (0·13); 3·90 (0·17)	3·86 (0·11); 3·90 (0·17)	3·60 (0·32); 3·73 (0·53)	2·93 (0·67); 2·97 (0·82)	2·40 (0·30); 2·40 (0·45)	36·26; <0·0001	0·40 (0·04)	0·0089 (0·45)	0·011 (0·58)	0·048 (0·46)	0·38
Texture level††	0·21 (0·21); 0·13 (0·23)	0·19 (0·18); 0·13 (0·25)	0·60 (0·50); 0·40 (0·73)	1·27 (0·64); 1·10 (0·78)	1·90 (0·44); 1·87 (0·73)	36·92; <0·0001	0·41 (0·03)	0·0084 (0·46)	0·025 (0·50)	0·028 (0·53)	0·38

Data are mean (SD); median (IQR) unless otherwise stated. COPD=chronic obstructive pulmonary disease. FEV_1_=forced expiratory volume in 1 s. FVC=forced vital capacity.

* H=9·49 is the critical H value for a confidence level of 0·95 in the Kruskal-Wallis test.

† p values calculated from the Mann-Whitney *U* test and effect size calculated by r=|z × n^−½^|; p<0·05 indicates a significant difference.

‡ For patients with FEV_1_/FVC<0·7 the results from after bronchodilator administration were used for the statistical analysis.

§ Percentage of the segmented lung that exhibits density values below −950 HU.

¶ Emphysema severity rating was obtained by calculating the mean over all five readers. For the parameters homogeneity level and texture level, the average over all six subregions and all readers was used for the evaluation.

‖ No evidence of emphysema (0), beginning of emphysema (1), mild emphysema (2), moderate emphysema (3), and severe emphysema (4).

** Very inhomogeneous (1), moderately inhomogeneous (2), mildly inhomogeneous or mostly homogeneous (3), and homogeneous (4).

†† No texture (0), subtle stains smaller than 5 mm (1), medium stains of 5–10 mm (2), and large stains bigger than 10 mm (3).

Consistently, patients graded with absent emphysema or trace emphysema in the visual CT evaluation were generally rated with lower severity levels based on the dark-field images. These severity ratings steadily increased with mild, moderate, confluent, and advanced destructive emphysema. Diagnostic confidence based on the dark-field images with respect to the severity classification was between certain and very certain. Larger variations of the diagnostic confidence were not found across the Fleischner groups (absent 3·6 [SD 0·3], trace 3·6 [0·2], mild 3·5 [0·3], moderate 3·7 [0·3], confluent and advanced destructive 3·9 [0·2]).

## Discussion

This study shows that dark-field chest radiography can technically and physically be translated to an application in humans using readily available x-ray imaging hardware and acceptable exposure doses. Furthermore, the first patient results confirm that x-ray dark-field chest imaging can detect structural impairments associated with COPD, which remains challenging using only conventional chest x-rays.

Because the lungs’ diffusion capacity strongly depends on their alveolar surface area, which in turn affects small-angle scattering of x-rays, the dark-field signal encodes spatially resolved information about lung health. This property assigns the dark-field signal a functional character (reflecting the integrity of the respiratory ability of the alveoli) in terms of chest imaging, which is confirmed by the good correlation with DLCO SB. However, these findings also indicate that the FEV_1_/FVC ratio cannot be associated entirely with the dark-field signal. A possible explanation might be the limited ability of pulmonary function testing to detect initial structural changes. Also, airflow limitation might indicate obstruction of the small airways without the presence of emphysema.^14^ Additionally, several groups have shown that many smokers without traditional spirometric obstruction have symptoms and structural changes (emphysema) similar to those seen in spirometrically diagnosed COPD.^31^, ^32^ In a study by Lynch and colleagues, some degree of emphysema at CT was found in 562 (44%) of 1285 smokers with no spirometric abnormality.^33^ Hence, it is not surprising to observe a gradual decrease of the dark-field signal in patients exhibiting a normal FEV_1_/FVC ratio.

By contrast to pulmonary function testing, dark-field radiography is an imaging technique that allows for a regional evaluation of the lungs’ structural condition. This feature might be useful for an improved treatment monitoring, phenotyping, or intervention planning.

Compared with the CT emphysema index, in which pathological classification is binary, the dark-field signal allows for a gradual assessment. This characteristic gave an improved capability to register the diffusion capacity in our study. Furthermore, the thresholding approach used to obtain the emphysema index is very sensitive to noise in the reconstructed images, because affected and healthy tissue exhibit similar density values.^34^, ^35^ For this reason, the differentiation of early disease stages using the CT emphysema index is limited.^33^ This limitation is also apparent in our findings, in which the Fleischner grades of trace, mild, and moderate emphysema yielded mean index values below the critical value of 6%.

Visual features (signal homogeneity and texture) observable in the dark-field images appear to provide greater diagnostic value than conventional emphysema characterising parameters when taking the Fleischner grading scheme as a reference standard.

In direct comparison, dark-field images and visual evaluation of CT images yield consistent findings regarding emphysema diagnosis. This finding is plausible considering that the Fleischner grade assignment is based on the size and distribution of regional density reduction originating from parenchymal impairments. The associated changes in the alveolar microstructure also induce local signal variations in the dark-field image due to reduced small-angle scattering in affected regions. In turn, the local signal variations affect the homogeneity and areal texture of the dark-field signal within the lung regions.

Drawing conclusions about the diagnostic performance of the dark-field images with respect to early disease stages based on this data is restricted as the reference standard itself might have limitations detecting initial changes. We find that the Fleischner scale does not deliver a significant correlation to the diffusion capacity if only emphysema grades from absent to mild are regarded, although the dark-field signal intensity still showed a moderate correlation for these groups in the examined collective. This finding might indicate an ambiguous interpretation of the true lung condition using CT at initial stages, limiting validation of diagnostic performance. This question must be addressed in a longitudinal study with a larger cohort.

The main limitation of this study is the relatively small number of included patients. The presented findings are preliminary and must be confirmed by a large, multi-ethnic cohort. A technical limitation is the 7 s breath hold during the acquisition, which might be challenging for patients with reduced lung function and requires further development. The presence of contrast agent during the CT examination is a systematic limitation in the measurement of emphysema. However, the presence of contrast agent should not induce a bias that compromises the conclusions of our study because the contrast agent is present across the entire group and so any change will be consistent across findings.

Emphysema-associated reduction of the respiratory surface has a negative effect on the lungs’ diffusion capacity and is generally irreversible. However, treatment options exist and can delay disease progression. Early disease detection is therefore crucial for an effective treatment and consequently an improved quality of life.^19^ At the same time, COPD and emphysema are highly underdiagnosed conditions.^31^, ^32^ Because of the absence of disease-modifying therapies, the role of COPD screening programmes, which could resolve this underdiagnosis, is controversial.^32^ With effective dose values considerably below those of low-dose CT (around 2%), the dark-field technique could contribute to an increased acceptance towards screening programmes. Providing detailed information on lung micromorphology, dark-field chest radiography could serve as a low-dose imaging tool enabling periodic examinations to help resolve the prevalent condition of underdiagnosed COPD. In the context of recently reported advances in regenerative therapeutics,^36^ dark-field imaging might support further translational research in this field and might at a later stage be integrated in disease management concepts for the monitoring of treatment response.

Currently, there is no commercial clinical dark-field system available. The clinical operation of our demonstrator system does not require specialised knowledge and is operationally comparable to conventional radiography systems. From an equipment perspective, we believe that x-ray dark-field radiography at this point of time is scalable to a broad application because the technique requires standard x-ray imaging components, which are already broadly available, and high-aspect-ratio microstructures, which have been optimised in the past 5 years allowing for a reproducible production and high quality. Because dark-field chest imaging is not limited to emphysema, further studies on other lung pathologies, such as fibrosis, pneumothorax, lung cancer, or pneumonia (including COVID-19 pneumonia) are of great interest.^8^, ^9^, ^11^, ^12^ Based on the reported results, we believe that x-ray dark-field chest imaging could contribute to improving the detection, diagnosis, and thus treatment and care of pulmonary disorders.

## Data sharing

After entering a signed data access agreement, underlying data and source code used in the evaluation of this study can be provided without patient identification upon reasonable request to researchers affiliated to accredited research institutions. Proposals are required to address scientific questions and will be reviewed individually. Please direct your request to the corresponding author (konstantin.willer@ph.tum.de).

## Declaration of interests

TK is an employee of Philips Innovative Technologies, Research Laboratories, which is part of Royal Philips. AY and TP are employees of Philips Medical System DMC, which is part of Royal Philips. GSZ receives payments for lectures from Boehringer Ingelheim, AstraZeneca, Novartis, GlaxoSmithKline, Bayer Healthcare, and Roche; and receives travel support from Novartis and Roche. FP received a European Research Council Advanced Grant and a hardware loan (x-ray source and detector) from Royal Philips. The authorship of patents related to technical implementation of the demonstrator system are KW has coauthored GB3687403B2 and US20200187893A1; FDM has coauthored GB3687403B2 and US20200187893A1; TU has coauthored PCT/EP2021/063949; TK has coauthored US10417761B2, US10945690B2, GB3687403B2, US10912532B2, PCT/EP2020/082799, PCT/EP2021/057524, PCT/EP2021/064497, PCT/EP2021/063949, and US20200187893A1; AY has coauthored GB3687403B2, US10912532B2, PCT/EP2020/082799, and US20200187893A1; TP has coauthored PCT/EP2020/082799; and FP has coauthored US10945690B2 and PCT/EP2021/063949. Royal Philips is the assignee for all patents listed. All other authors declare no competing interests.

## Supplementary Materials

Supplementary appendix

Supplementary Video Example video sequence of a patient scan The sequence comprises raw images of an actual patient scan and was generated from the raw pixel data as obtained from the flat-panel detector. The acquisition frequency (30 Hz) was set as the frame rate of the video, playing back the acquisition in real time. To retrieve the conventional attenuation and dark-field images, the moiré pattern overlaying the patient's thorax is mapped onto a sinusoidal model in subsequent processing steps. At the contour of the heart, slight displacements induced by the heartbeat can be recognised. The scan video shows the acquisition of the patient presented in figure 1B–D in the Article. YouTube link: https://youtu.be/lAL7R0uhuvg

## References

[1] Pfeiffer F, Bech M, Bunk O (2008). Hard-x-ray dark-field imaging using a grating interferometer. Nat Mater.

[2] Schleede S, Meinel FG, Bech M (2012). Emphysema diagnosis using x-ray dark-field imaging at a laser-driven compact synchrotron light source. Proc Natl Acad Sci USA.

[3] Yaroshenko A, Meinel FG, Bech M (2013). Pulmonary emphysema diagnosis with a preclinical small-animal x-ray dark-field scatter-contrast scanner. Radiology.

[4] Bech M, Tapfer A, Velroyen A (2013). In-vivo dark-field and phase-contrast x-ray imaging. Sci Rep.

[5] Bech M, Schleede S, Potdevin G (2011). Experimental validation of image contrast correlation between ultra-small-angle x-ray scattering and grating-based dark-field imaging using a laser-driven compact x-ray source. Photonics Lasers Med.

[6] Meinel FG, Yaroshenko A, Hellbach K (2014). Improved diagnosis of pulmonary emphysema using in vivo dark-field radiography. Invest Radiol.

[7] Hellbach K, Yaroshenko A, Meinel FG (2015). In vivo dark-field radiography for early diagnosis and staging of pulmonary emphysema. Invest Radiol.

[8] Yaroshenko A, Hellbach K, Yildirim AÖ (2015). Improved in vivo assessment of pulmonary fibrosis in mice using x-ray dark-field radiography. Sci Rep.

[9] Hellbach K, Yaroshenko A, Willer K (2016). Facilitated diagnosis of pneumothoraces in newborn mice using x-ray dark-field radiography. Invest Radiol.

[10] Yaroshenko A, Pritzke T, Koschlig M (2016). Visualization of neonatal lung injury associated with mechanical ventilation using x-ray dark-field radiography. Sci Rep.

[11] Scherer K, Yaroshenko A, Bölükbas DA (2017). X-ray dark-field radiography – in-vivo diagnosis of lung cancer in mice. Sci Rep.

[12] Hellbach K, Meinel FG, Conlon TM (2018). X-ray dark-field imaging to depict acute lung inflammation in mice. Sci Rep.

[13] WHO (2018). The top 10 causes of death. https://www.who.int/news-room/fact-sheets/detail/the-top-10-causes-of-death.

[14] Labaki WW, Martinez CH, Galbàn CG (2017). The role of chest CT in the evaluation and management of patients with chronic obstructive pulmonary disease. Am J Respir Crit Care Med.

[15] Han MK (2018). Should chest CT be part of routine clinical care for COPD? No. Chest.

[16] Mettler FA, Huda W, Yoshizumi TT, Mahesh M (2008). Effective doses in radiology and diagnostic nuclear medicine: a catalog. Radiology.

[17] Larke FJ, Kruger RL, Cagnon CH (2011). Estimated radiation dose associated with low-dose chest CT of average-size participants in the National Lung Screening Trial. AJR Am J Roentgenol.

[18] Pratt PC (1987). Role of conventional chest radiography in diagnosis and exclusion of emphysema. Am J Med.

[19] Global Initiative for Chronic Obstructive Lung Disease (2019). Global strategy for the diagnosis, management, and prevention of chronic obstructive pulmonary disease 2019 report. https://goldcopd.org/wp-content/uploads/2018/11/GOLD-2019-v1.7-FINAL-14Nov2018-WMS.pdf.

[20] Pfeiffer F, Weitkamp T, Bunk O, David C (2006). Phase retrieval and differential phase-contrast imaging with low-brilliance x-ray sources. Nat Phys.

[21] Gromann LB, De Marco F, Willer K (2017). In-vivo x-ray dark-field chest radiography of a pig. Sci Rep.

[22] Hellbach K, Baehr A, De Marco F (2018). Depiction of pneumothoraces in a large animal model using x-ray dark-field radiography. Sci Rep.

[23] Willer K, Fingerle AA, Gromann LB (2018). X-ray dark-field imaging of the human lung—a feasibility study on a deceased body. PLoS One.

[24] De Marco F, Willer K, Gromann LB (2019). Contrast-to-noise ratios and thickness-normalized, ventilation-dependent signal levels in dark-field and conventional in vivo thorax radiographs of two pigs. PLoS One.

[25] Sauter AP, Andrejewski J, De Marco F (2019). Optimization of tube voltage in x-ray dark-field chest radiography. Sci Rep.

[26] Fingerle AA, De Marco F, Andrejewski J (2019). Imaging features in post-mortem x-ray dark-field chest radiographs and correlation with conventional x-ray and CT. Eur Radiol Exp.

[27] Coates AL, Peslin R, Rodenstein D, Stocks J (1997). Measurement of lung volumes by plethysmography. Eur Respir J.

[28] Wanger J, Clausen JL, Coates A (2005). Standardisation of the measurement of lung volumes. Eur Respir J.

[29] Lynch DA, Austin JHM, Hogg JC (2015). CT-definable subtypes of chronic obstructive pulmonary disease: a statement of the Fleischner Society. Radiology.

[30] Johns DP, Walters JAE, Walters EH (2014). Diagnosis and early detection of COPD using spirometry. J Thorac Dis.

[31] Regan EA, Lynch DA, Curran-Everett D (2015). Clinical and radiologic disease in smokers with normal spirometry. JAMA Intern Med.

[32] Martinez CH, Mannino DM, Jaimes FA (2015). Undiagnosed obstructive lung disease in the United States – associated factors and long-term mortality. Ann Am Thorac Soc.

[33] Lynch DA, Moore CM, Wilson C (2018). CT-based visual classification of emphysema: association with mortality in the COPDGene study. Radiology.

[34] Gierada DS, Bierhals AJ, Choong CK (2010). Effects of CT section thickness and reconstruction kernel on emphysema quantification relationship to the magnitude of the CT emphysema index. Acad Radiol.

[35] den Harder AM, de Boer E, Lagerweij SJ (2018). Emphysema quantification using chest CT: influence of radiation dose reduction and reconstruction technique. Eur Radiol Exp.

[36] Conlon TM, John-Schuster G, Heide D (2020). Inhibition of LTβR signalling activates WNT-induced regeneration in lung. Nature.

