# Supplementary material for: X-ray dark-field chest imaging for detection and quantification of emphysema in patients with chronic obstructive pulmonary disease: a diagnostic accuracy study
Source: Lancet Digit Health. 2021 Oct 25;3(11):e733–44. doi: 10.1016/S2589-7500(21)00146-1 (PMC8565798; doi:10.1016/S2589-7500(21)00146-1)
Supplement: Supplementary appendix [file mmc1.pdf]

THE LANCET
Digital Health

Supplementary appendix

This appendix formed part of the original submission and has been peer reviewed. We post it as supplied by the authors.

Supplement to: Willer K, Fingerle AA, Noichl W, et al. X-ray dark-field chest imaging for detection and quantification of emphysema in patients with chronic obstructive pulmonary disease: a diagnostic accuracy study. *Lancet Digit Health* 2021; **3:** e733–44.

Supplementary appendix

The online material provides further information on our demonstrator system, CT evaluation, and statistical data analysis. Furthermore, we present the study results of two additional participants with opposite smoking habits, one without pulmonary impairment and one with severe emphysematous destruction.

Chest X-ray dark-field demonstrator system

Hardware. The design of the demonstrator system is based on standard medical components, such as X-ray source, collimator, and flat-panel detector, which are combined with a three-grating Talbot-Lau X-ray interferometer.[1,2] A sketch of the system is depicted in figure 1A of the main manuscript. Via phase modulation of the X-ray beam, the grating G_1 generates a high-frequency intensity pattern with a periodicity of a few microns at the position of the grating G_2 that consists of highly absorbing gold lines. By utilizing a similarly high spatial frequency in the G_2 structure, this pattern is down-converted to low-frequency moiré fringes with a period of a few millimeters, which can be recorded conveniently by conventional medical imaging detectors. Finally, the grating G_0 is necessary to allow the use of high-power medical X-ray sources, which typically have rather large focal spot sizes (i.e. several hundred microns). The installation of G_0 close to the source ensures that every individual beamlet coming from a gap in G_0 has sufficient transverse coherence of X-rays, necessary for the formation of the high-frequency intensity pattern.[1]
If an object with a large number of inherent interfaces on the micron scale (e.g. lung tissue) is placed within this arrangement, the contrast (visibility) of the moiré pattern decreases as a result of multiple refractions on these interfaces.[2–4] This allows assessing characteristics of structures significantly smaller than the resolution limit of the imaging system.[2] The magnitude of the dark-field signal is encoded in the reduced visibility V of the moiré pattern, whereas the magnitude of the attenuation signal is given by the pattern's decreased mean intensity I.
The high-aspect-ratio grating structures were manufactured utilizing X-ray lithography and subsequent gold electroplating (microworks GmbH, Germany). Due to current limitations in grating fabrication, typical sizes currently achieved with this technique range in the order of some centimeters. To extend the coverage suitable to image an entire human thorax, a combination of grating tiling[5] and interferometer scanning[6,7] was established for the demonstrator system. As it is the grating most distant from the source, it is the analyzer grating G_2 that limits the size of the active area usable for acquisition. It is assembled from six individual grating tiles ($6\times7{\cdot}0\times6{\cdot}5$ cm^2) to entirely cover the field of view in horizontal direction. By moving the interferometer relative to the imaging system during acquisition, the desired coverage in the vertical direction is achieved. This results in a total field of view of 37×37 cm^2 with respect to the patient plane. In addition, relative phase shifts between analyzer grating and the high-frequency intensity pattern generated by G_1 were encoded in the vertical coordinate of the active area. By that, a sinusoidal data set carrying attenuation and dark-field information can be sampled within one pixel during the scan movement. The relative shifts arise in the form of the aforementioned moiré fringes, which are depicted in figure S1 by an exemplary single exposure without patient. Over the entire field of view, we obtain a mean visibility of about 31%.

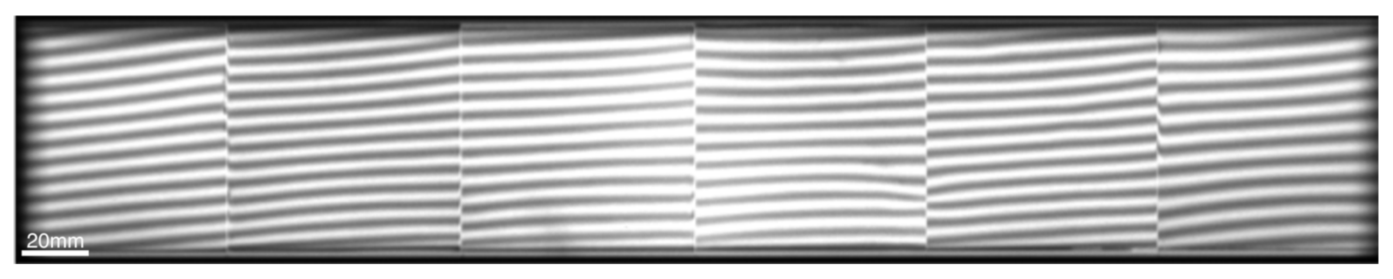

Figure S1: Single raw exposure without patient. The G_2 is stitched together from six individual high aspect ratio gold grating tiles forming the active area of about 42x6·5 cm^2. With 0·5 mm, the gap size between the tiles slightly exceeds the image pixel size. By scanning the active area in vertical direction, a field of view of about 37x37 cm^2 with respect to the patient plane is achieved. The moiré fringes serve as sampling pattern for the attenuation and the dark-field signal.

In order to avoid shadowing induced by the binary grating structures, all gratings are adapted to the divergent beam shape by cylindrical bending with respect to the corresponding distance to the focal spot of the X-ray source. In addition, the scan movement is performed following an arc, also centered on the focal spot, to preserve the incident beam angle onto the gratings throughout the scan. The grating arrangement is mounted on an aluminum frame construction that is attached to a spindle drive performing the scan movement. The connection to the drive is realized as a sliding hinge to provide the required degrees of freedom for the rotational scan movement.
After a short acceleration phase, as soon as the active area reaches the field of view of the imaging system, the source (MRC 200 0508, Royal Philips, The Netherlands) is triggered to emit X-ray pulses with a repetition rate of 30 Hz. Simultaneously, the flat-panel detector (Pixium FE 4343, Trixell, France) acquires images with an exposure time of about 17 ms per frame. If utilizing the maximal achievable field of view, a total of 195 frames are acquired. A video sequence (video 1) playing back raw detector images of an actual patient scan in real time is available online.

Radiation dose. In order to minimize radiation exposure, our system was designed with specific focus on dose efficiency. An inverse interferometer arrangement[8] was selected to avoid the G_1 grating acting as an additional absorber downstream of the patient position. Secondly, a combination of a stationary (MTR 302, Ralco, Italy) and movable (2 mm Tungsten) collimator was used to avoid exposure in regions that are not covered by the gratings and hence do not contribute to the image. After moving the interferometer out of the beam path, the stationary collimator can be adjusted according to the entire region to be imaged. This is achieved with motorized collimator blades that are illuminated for visual position feedback. The movable collimator is attached to the interferometer and restricts the beam to the grating area. An additional measure for minimizing patient exposure is synchronizing detector readout and exposure to avoid dose deposition during insensitive time intervals. Further, intrinsic beam filtration (2·5 mm Aluminum) removes low-energy photons that would not penetrate the patient and thus would not contribute to image formation.
Downstream of the two collimation layers covering the entire radiation area, an ionization air-chamber (Diamentor CI, PTW, Germany) is mounted to log the applied radiation dose of every scan. Via a measurement of the dose area product, the effective patient dose applied during one scan is estimated. The respective conversion coefficient (1·5 μSv/μGy·m^2) was determined utilizing an anthropomorphic thorax phantom (ATOM 701, CIRS, USA) with embedded thermoluminescent dosimeters. The phantom simulates X-ray attenuation properties of a male reference person with a body size and weight of 173 cm and 73 kg. With respect to such a reference person undergoing one scan in posterior-anterior orientation, the received effective dose was determined to be 0·035 mSv. To ensure a constant dose in the image receptor plane (~3·5 μGy, behind the lungs), the tube current is modified according to the patient's size and weight. This ensures a constant image quality over the patient collective, but also implies that patients with a higher BMI receive a higher radiation dose. The acceleration voltage was set to 70 kV_p, which is a reasonable compromise between interferometer performance and X-ray transmission.[9]

Data processing. Both modalities, conventional attenuation and dark-field, are jointly calculated from the data acquired within a single scan, yielding two perfectly registered images. The data processing essentially relies on the approach described in ref.[7], extended by additional correction and calibration procedures to account for signal corruption induced by Compton scattering and beam hardening. The moiré pattern in each pixel of the raw images is mapped onto a sinusoidal intensity model, which allows the extraction of the pattern's mean value I (e.g. figure 1C: conventional attenuation) and visibility V (e.g. figure 1D: dark-field). Both quantities are normalized to air scan values (I_0, V_0) to account for inhomogeneities ranging over the field of view. As intensity and visibility decline exponentially with object thickness,[10] both signals are given as the negative natural logarithm of the respective normalized quantity, i.e.: $-\ln(I/I_0)$ and $-\ln(V/V_0)$.

Corrections and signal calibrations. In addition to an air scan normalization, corrections on the dark-field and attenuation images are applied in order to account for Compton scattering and beam hardening.
Secondary photons that originate from Compton scatter can be considered as diffuse illumination that increases the mean value of the fringe pattern, but not its amplitude. This results in a decreased visibility and thus has a negative influence on the measurement of the dark-field signal. Although the high-aspect-ratio G_2 grating already removes a majority of these photons, a significant part, primarily scattered in a direction parallel to the grating lamellae, still reaches the detector. To account for this effect, a commercially available software (SkyFlow, Royal Philips, The Netherlands) for the estimation of Compton scattering, based on Monte Carlo simulations and a preliminary attenuation image, was adapted to the dark-field demonstrator system and is utilized to correct the raw images before data processing.
The beam hardening correction is applied to the dark-field images to account for spectral differences between patient and reference scan[11]. Hereby, calibration data obtained from a series of visibility measurements with a varying number of tissue-equivalent absorbing sheets introduced into the beam path is used to correct the air-scan visibility. The attenuation image is used to generate a correction map, based on the assumption of similar modification of the X-ray spectrum in the calibration and the patient scan.
Image post-processing including histogram adaptation and edge enhancement using a commercially available software (UNIQUE, Royal Philips, The Netherlands) is performed on the attenuation image to obtain an image quality comparable to conventional systems. The data given in Table 1 was extracted prior to this post-processing.
In the dark-field image, pixel lines coinciding with tiling gaps (~0·5 mm) are replaced by interpolation between neighboring pixels. This step can be omitted for the attenuation signal as the gaps are corrected by the air scan normalization.
Determining the illumination during the scan, the collimated active area moves across one pixel in roughly 0·8 sec. Tissue displacement during this time interval, in particular occurring in the heart and diaphragm region, can induce artefacts in the reconstructed images. To reduce these artefacts, a correction algorithm is applied that is based on virtually curtailing the sampling of affected regions.
In order to obtain uniform noise variance independent from the sample's transmission, adaptive denoising is applied to the dark-field image.

Clinical operation. The operation of the system is comparable to the one of a conventional medical radiography system. Prior to the scan, the radiographer explains the procedure to the patient and adjusts the field of view as well as the patient's position. After moving the interferometer to the starting position and leaving the examination room, the operator can trigger the acquisition via a control panel. For the duration of the scan, the patient remains positioned in the optical path of the scanner, holding breath. To properly synchronize breath-hold and acquisition, the patient obtains automatically triggered audio commands during the acceleration phase of the interferometer and after the acquisition. In order to reduce displacement artifacts, the scan is performed in upwards direction, as premature breathing (if prevalent) is expected to be more likely to occur towards the end of the acquisition. At that time, the active grating area has already passed the diaphragm region where larger shifts of tissue are expected.

Quantitative CT evaluation

Figure S2 shows screenshots of the CT-based emphysema evaluation software (IntelliSpace, Royal Philips, The Netherlands) for the patient depicted in figure 2 of the main manuscript. The histogram illustrates the density distribution within the lung region. All voxels featuring density values below the threshold are assigned to be emphysematous, 11·5% in this case. The upper-lobe impairment predominance revealed in the dark-field image (figure 2B) is also indicated by the sectional evaluation by lung lobe: Right upper lobe (RUL): 21·1%; right middle lobe (RML): 13·7%; right lower lobe (RLL): 3·5%; left upper lobe (LUL): 14·3%; left lower lobe (LLL): 3·4%.

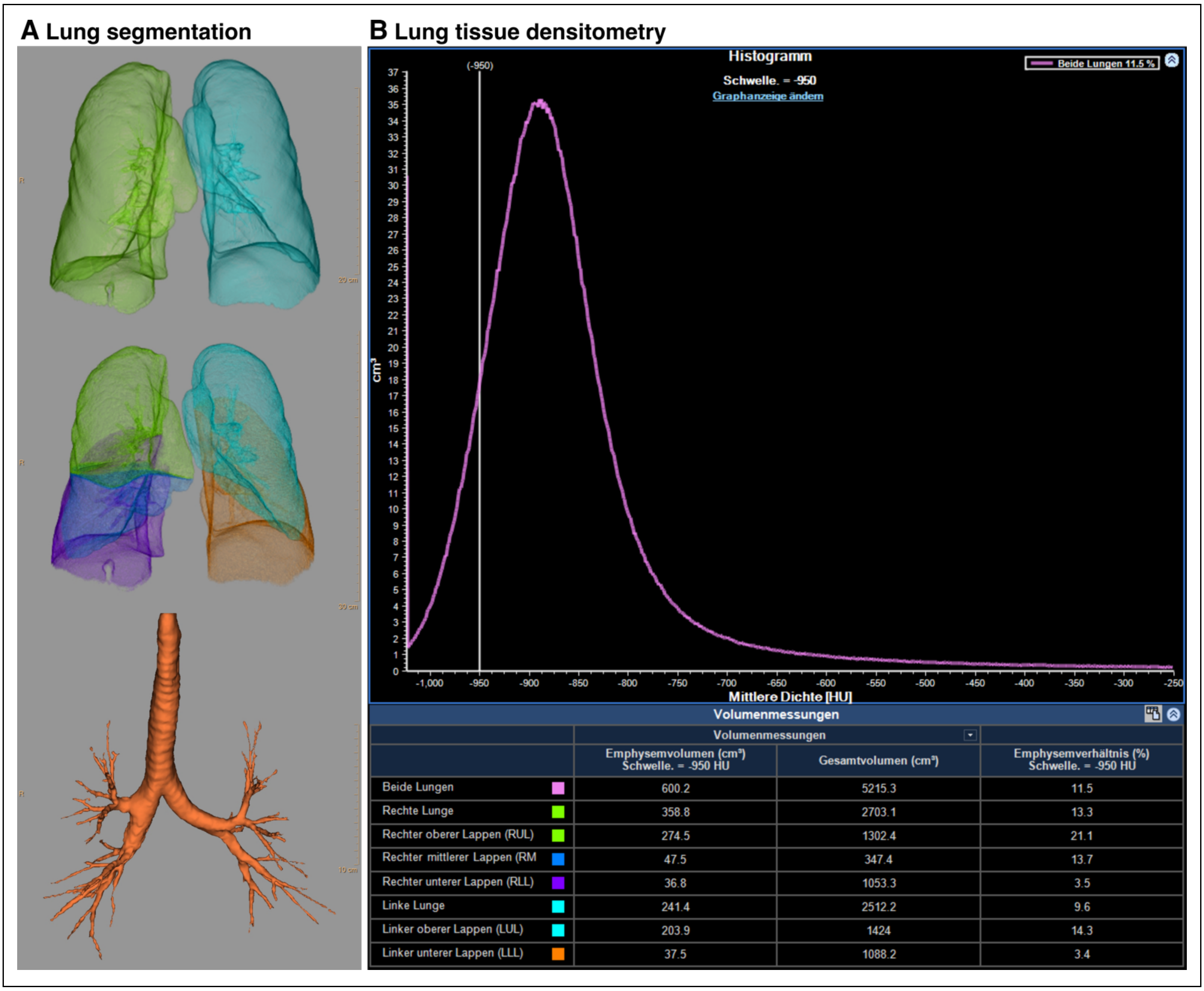

Figure S2: Screenshots of CT-based emphysema evaluation for the patient presented in figure 2. (A) shows the result of the segmentation of lungs, lobes, and bronchi. In (B), the density distribution within the segmented lung and a detailed listing of the emphysema index (rightmost table column), subdivided into lung side and lobes are depicted. The vertical line in the histogram denotes the threshold (-950 HU) for the evaluation. In the present case, 11·5% of the entire lung volume is classified as emphysematous.

Signal measurement

In order to measure the mean attenuation and dark-field signal intensity within the lung region and respective subregions (upper, middle, and lower of left and right lung), an individual mask for every patient was generated by manually selecting the outline of the lungs and subsequently calculating the average signal value of all included pixels. Lung regions superimposed by the heart and the diaphragm were omitted in order to exclude size-specific contributions from these parts. The mask was split into three parts of approximately equal height between apex and costodiaphragmatic recess to obtain the subregions of the reader study for each lung. The individual lung masks were generated on the basis of the attenuation images due to indistinct course of the lung outline in the dark-field image of patients with emphysema induced signal reduction (e.g. figure 2B of the main manuscript). However, as both signal channels originate from the same data set, the pixel information is perfectly registered, and the mask coordinates can be transferred directly to the dark-field channel. Exemplarily, figure S3 shows the respective mask for the patient depicted in figure 1B–D of the main manuscript. For the mask generation, calculations of the correlation coefficients, and data handling, open-source software frameworks (NumPy, PIL, matplotlib, SciPy, Pandas, Python) were utilized.

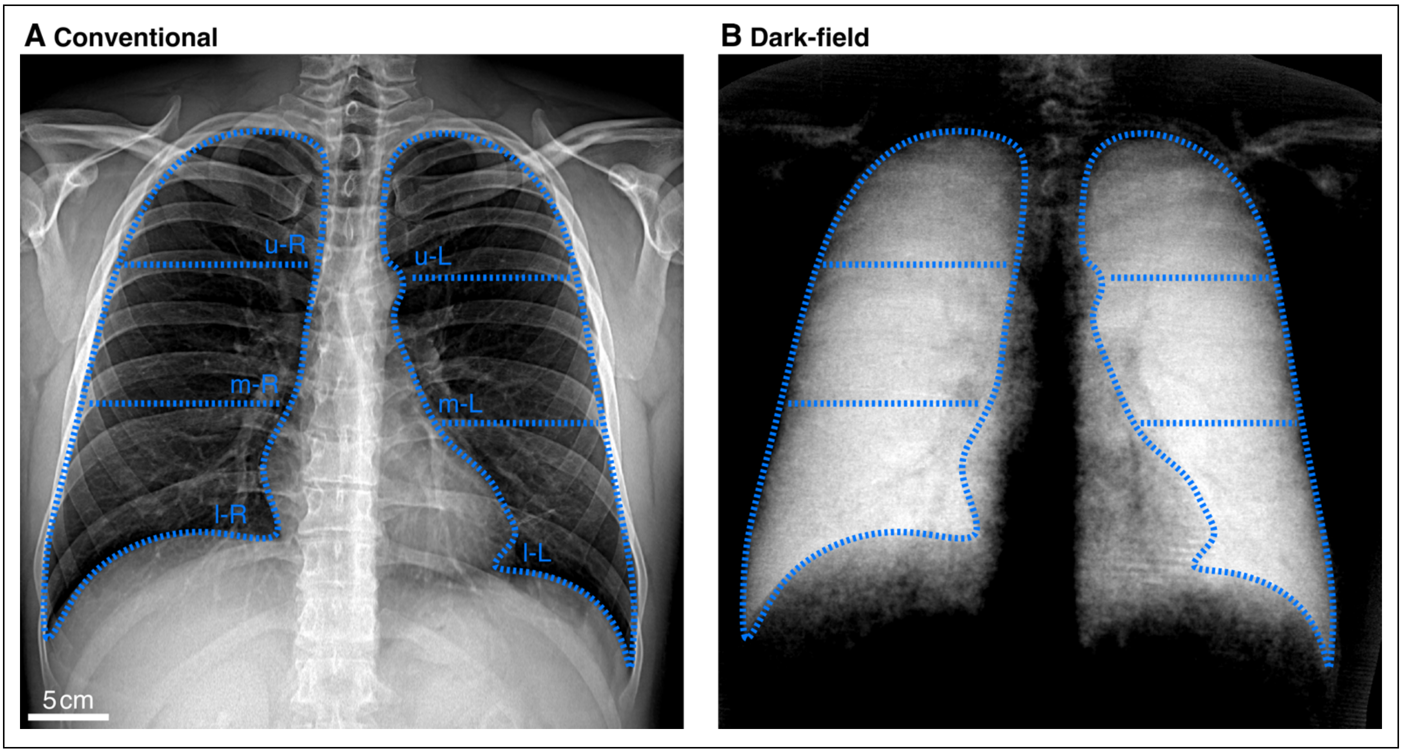

Figure S3: Exemplary lung masks generated manually for the statistical evaluation. Conventional (A) and dark-field (B) X-rays of the patient depicted in Figure 1 with superimposed lung masks. The masks were generated by manually tracing the boundary of the lungs in the attenuation image and splitting each lung into an upper, middle, and lower part. Heart and diaphragm regions were omitted. In order to obtain a reference measure for comparison with the reader ratings, the average signal from all coordinates lying within the boundary was calculated.

Subject recruitment

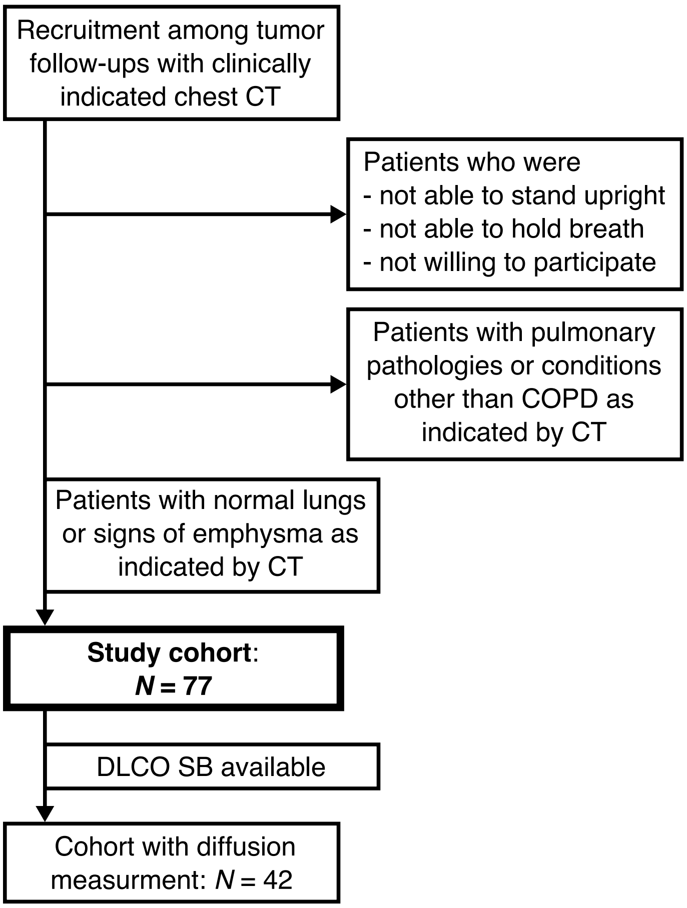

Figure S4: Flowchart illustrating subject recruitment. Between October 2018 and December 2019, a total number of 77 subjects were included in the study.

Additional cases

In the following, we present two additional cases from our study. Figure S5 provides an overview of all study data for a patient (male, 71 years, BMI 29·2) without smoking history. The attenuation-based radiograph (figure S5A) provides no conspicuous findings with regard to pulmonary emphysema, and the air-flow curve as well as the spirometric parameters (figure S5C) indicate normal pulmonary function. No secondary measurement with prior bronchodilator administration was performed, as no signs suggesting pulmonary obstructions were prevalent. The dark-field image (figure S5B) exhibits a homogeneous signal over the entire lung region. With a mean value of 0·28, however, the signal average is slightly reduced compared to those obtained for younger, healthy patients. This slight reduction also appears to be implied by the findings in CT densitometry (figure S5D) indicating a slightly elevated volume fraction (3·9%), with lower density values distributed homogeneously over the entire lung.

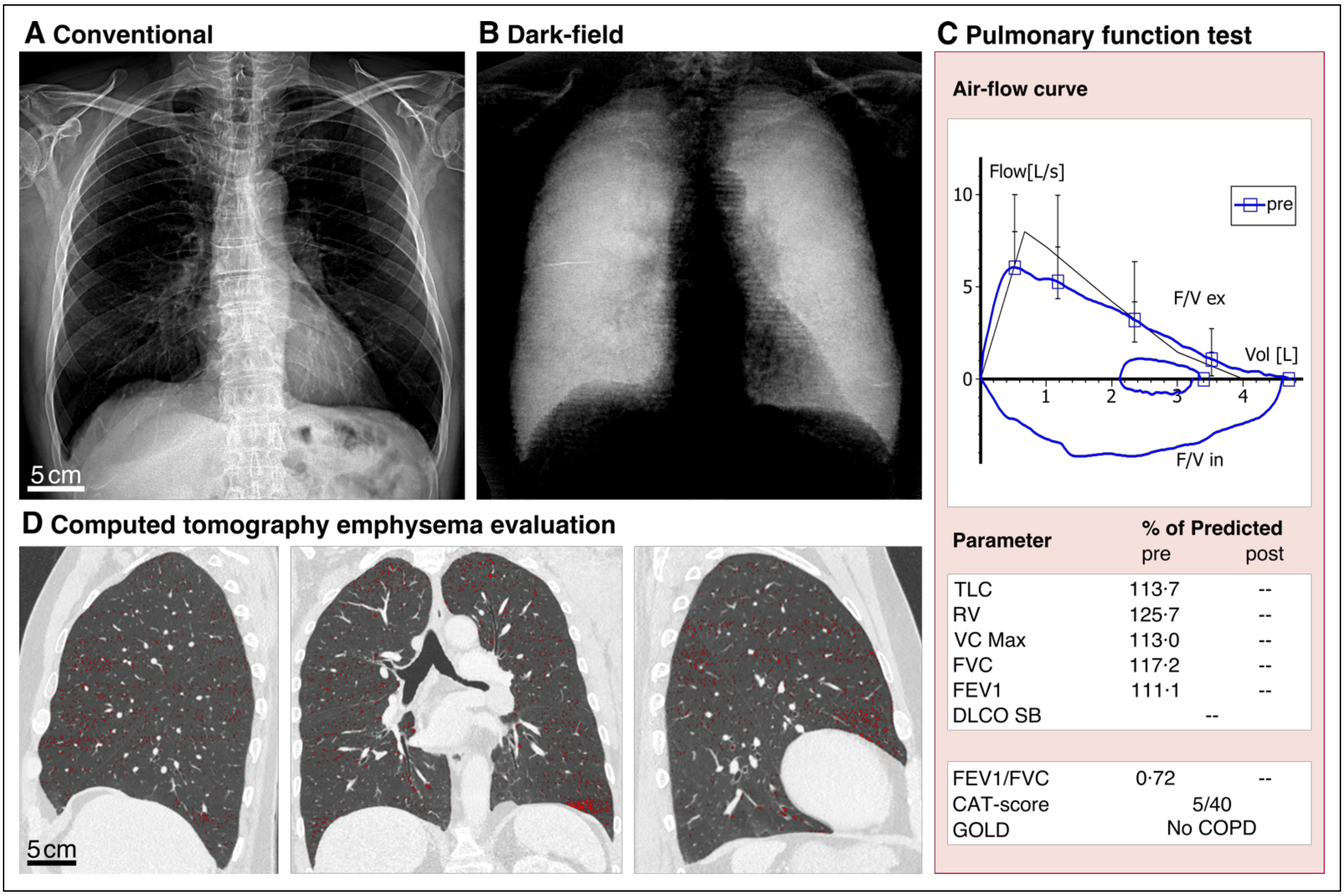

Figure S5: Clinical study results of a patient without COPD. Conventional attenuation (A) and dark-field (B) images of a patient (male, 71 years, BMI 29·2, non-smoker) without clinical indications of COPD. The dark-field image shows a homogeneous signal distribution over the lung with a slightly decreased mean value of 0·28, which might be an indication for a beginning impairment of the respiratory surface. For the attenuation signal, an average value over the entire lung of 2·74 was measured. With an inconspicuous air-flow curve and normal spirometric parameters (C), a healthy condition of the lung is indicated. CT densitometry yields an emphysema ratio of 3·9%, with detected areas being finely distributed over the entire lung, as depicted in the CT slices (D) where corresponding pixels are marked in red.

The second patient (male, 65 years, BMI 21·0) is a heavy smoker with a history of 80 pack-years, suffering from severe pulmonary emphysema. In figure S6, the respective study results are depicted. The attenuation image (figure S6A) shows enlarged intercostal spaces, a long barrel thorax shape including an elongated heart region, and a flat diaphragm, which all are advanced-stage, secondary symptoms of an emphysema. No significant contrast variation is observable. Indicating a substantial reduction of respiratory surface, low dark-field signal values (figure S6B) are obtained over the entire lung region. Towards apical regions appearing with inhomogeneous signal texture, a stronger manifestation of emphysematous destruction is revealed. Classifying the patient in GOLD-group 4, spirometry (figure S6C) yields a highly obstructed pulmonary condition that is preserved after bronchodilation. Substantial impairment is also reflected by a high volume-fraction (26%) featuring reduced density values in the CT reconstruction. Similar to the dark-field radiographs, a stronger emphysema manifestation can be identified in upper lung regions in the CT images (figure S6D). The regional prevalence of reduced density in the CT images is in good accordance with the signal distribution in the dark-field image.

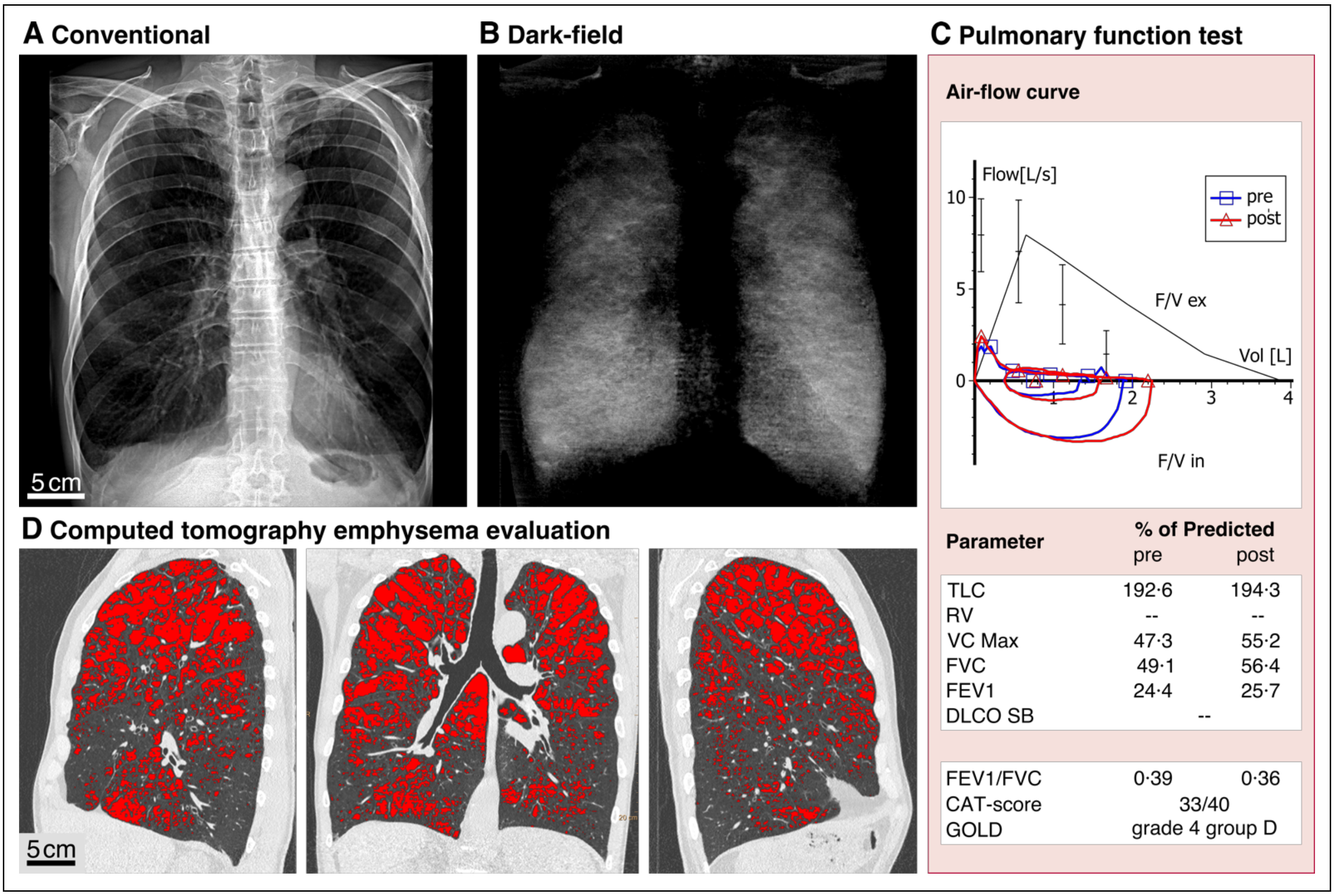

Figure S6: Study results of a patient graded with GOLD stage 4. Conventional attenuation (A) and dark-field (B) images of a patient (male, 65 years, BMI 21·0, 80 pack-years) suffering from severe emphysema affecting the entire lung, with stronger manifestation in upper regions. Reduced dark-field values indicate a degraded alveolar network with a decreased number of air-tissue interfaces. Over the entire pulmonary region, a mean value of 0·07 was measured for the dark-field and 2·31 for the attenuation. Spirometry (C) yields an obstructive condition of the lung with strongly deviating parameters that are preserved after administration of a bronchodilator. CT densitometry yields an emphysema index of 26%. The corresponding voxels in the CT slices (D) are marked in red. Dark-field and CT images yield consistent information with regard to parenchymal condition. Note that, this patient was not included in the statistical evaluation, as he underwent a native CT examination.

Caption for video 1

Video 1: Exemplary video sequence of a patient scan. The sequence comprises raw images of an actual patient scan and was generated from the raw pixel data as obtained from the flat-panel detector. The acquisition frequency (30 Hz) was set as frame rate of the movie, playing back the acquisition in real time. In order to retrieve the conventional attenuation and dark-field images, the moiré pattern overlaying the patient's thorax is mapped onto a sinusoidal model in subsequent processing steps. At the contour of the heart, slight displacements induced by the heartbeat can be recognized. The scan movie shows the acquisition of the patient presented in figure 1B–D of the main manuscript.

References (Appendix)

1. Pfeiffer F, Weitkamp T, Bunk O, David C. Phase retrieval and differential phase-contrast imaging with low-brilliance X-ray sources. *Nat Phys* 2006; **2**: 258–61.
2. Pfeiffer F, Bech M, Bunk O, et al. Hard-X-ray dark-field imaging using a grating interferometer. *Nat Mater* 2008; **7**: 134–37.
3. Schleede S, Meinel FG, Bech M, et al. Emphysema diagnosis using X-ray dark-field imaging. *Proc Natl Acad Sci* 2012; **109**: 17880–85.
4. Bech M, Tapfer A, Velroyen A, et al. In-vivo dark-field and phase-contrast X-ray imaging. *Sci Rep* 2013; **3**: 3209.
5. Schröter TJ, Koch FJ, Meyer P, et al. Large field-of-view tiled grating structures for X-ray phase-contrast imaging. *Rev Sci Instrum* 2017; **88**: 015104.
6. Kottler C, Pfeiffer F, Bunk O, Grünzweig C, David C. Grating interferometer based scanning setup for hard X-ray phase contrast imaging. *Rev Sci Instrum* 2007; **78**: 043710.
7. Koehler T, Daerr H, Martens G, et al. Slit-scanning differential X-ray phase-contrast mammography: proof-of-concept experimental studies. *Med Phys* 2015; **42**: 1959–65.
8. Donath T, Chabior M, Pfeiffer F, et al. Inverse geometry for grating-based X-ray phase-contrast imaging. *J Appl Phys* 2009; **106**: 054703.
9. Sauter AP, Andrejewski J, De Marco F, et al. Optimization of tube voltage in X-ray dark-field chest radiography. *Sci Rep* 2019; **9**: 8699.
10. Bech M, Schleede S, Potdevin G, et al. Experimental validation of image contrast correlation between ultra-small-angle X-ray scattering and grating-based dark-field imaging using a laser-driven compact X-ray source. *Photon Laser Med* 2011; **1**: 47–50.
11. Chabior M, Donath T, David C, et al. Beam hardening effects in grating-based X-ray phase-contrast imaging. *Med Phys* 2011; **38**: 1189-95.
